# Incidence of Post-dural Puncture Headache and Low Backache Following Median Versus Paramedian Spinal Anesthesia: A Randomized Double-Blind Trial

**DOI:** 10.7759/cureus.98172

**Published:** 2025-11-30

**Authors:** Shreya Dutta, Laxman Kumar Senapati, Ayesha Pattnaik, Partha S Mohapatra

**Affiliations:** 1 Department of Anesthesiology, Kalinga Institute of Medical Sciences, Bhubaneswar, IND

**Keywords:** lower segment cesarean section, median, paramedian, post-dural puncture headache, post-spinal backache, spinal anesthesia

## Abstract

Background and aim

Spinal anesthesia (SA) is widely used for cesarean sections, but complications such as post-dural puncture headache (PDPH) and post-spinal backache (PSBA) remain common. The two principal techniques, the median and paramedian approaches, differ in anatomical pathways and potential tissue trauma. This study aimed to compare the incidence and severity of PDPH and PSBA following SA via the median versus paramedian approach in parturients undergoing elective lower-segment cesarean section (LSCS).

Methodology

This randomized, double-blind trial was conducted in 166 American Society of Anesthesiologists (ASA) II-III parturients aged 18-40 years scheduled for elective LSCS. They were randomly assigned to receive SA via either the median (group M, n=83) or paramedian (group P, n=83) approach using a 25 G Quincke needle and 2.2 mL of 0.5% hyperbaric bupivacaine. Participants were monitored for PDPH at 24 h, 72 h, and one week, and for PSBA at one week, one month, and three months following surgery. Pain intensity was assessed using the visual analog scale (VAS). Data were analyzed using SPSS version 26 (Armonk, NY: IBM Corp.), with p<0.05 considered statistically significant.

Results

Baseline demographics were comparable between groups. The incidence of PDPH was significantly lower in the paramedian group compared with the median group at 24 h (13 versus 38; p=0.001), 72 h (10 versus 22; p=0.029), and one week (5 versus 15; p=0.030). PSBA was also lower in the paramedian group at one week (16 versus 29; p=0.035), though differences at one month and three months were not statistically significant. Pain severity (VAS) scores for both PDPH and PSBA were generally lower in the paramedian group throughout follow-up.

Conclusion

The paramedian approach to SA in elective LSCSs was associated with a consistently lower incidence and severity of PDPH and PSBA compared to the median approach. These findings suggest that the paramedian technique may enhance post-operative comfort and could be preferred, particularly in patients with complex spinal anatomy or higher PDPH risk.

## Introduction

Spinal anesthesia (SA) is a widely used regional technique for lower abdominal, pelvic, and lower-limb surgeries, including elective cesarean sections. It offers rapid onset, dense neural blockade, minimal systemic effects, and allows maternal awareness and early bonding with the newborn. Over the years, two principal needle insertion techniques - the median and paramedian approaches - have remained central to clinical practice, each offering advantages depending on patient anatomy and operator preference [1,2].

The median approach, with midline entry between the spinous processes, is traditionally taught and commonly used. However, it may be technically challenging in patients with obesity, difficult surface landmarks, or degenerative spinal changes [3-5]. In such situations, the paramedian approach, which inserts the needle slightly lateral to the midline while avoiding the supraspinous and interspinous ligaments, may facilitate easier access and potentially reduce soft-tissue trauma [6].

Post-dural puncture headache (PDPH) remains one of the most common complications of SA. It is attributed to cerebrospinal fluid (CSF) leakage through the dural defect, resulting in intracranial hypotension and orthostatic headache [7]. Reported PDPH incidence in obstetric populations ranges widely, from 1% to 14%, depending on needle type, gauge, orientation, and insertion technique [8]. Some studies suggest that oblique or lateral needle trajectories, as in the paramedian approach, may create a more slit-like dural defect and reduce CSF leakage, though the evidence remains mixed [9,10].

Post-spinal backache (PSBA) is another recognized but typically self-limiting morbidity after SA. It arises from local soft-tissue trauma, patient positioning, or ligamentous strain, and may influence early mobilization and maternal satisfaction [11,12]. As with PDPH, PSBA incidence may vary by technique, with some reports indicating lower rates when midline ligamentous structures are avoided [13,14].

Given these potential differences, comparing the two approaches in obstetric patients is clinically relevant. Therefore, this randomized, double-blind study was designed to compare the incidence and visual analog scale (VAS)-based severity of PDPH and PSBA between the median and paramedian approaches in low- to moderate-risk obstetric patients undergoing elective cesarean delivery. We hypothesized that the paramedian approach would be associated with a lower incidence and severity of PDPH and PSBA, as measured by VAS, compared with the median approach. The primary objective of this study was to compare the incidence and visual analog scale (VAS)-based severity of PDPH and post-spinal backache between the median and paramedian approaches in low- to moderate-risk obstetric patients. The secondary objectives encompassed evaluating PDPH severity via the visual analog scale (VAS), examining the incidence and severity of PSBA at one week, one month, and three months post-operatively, and comparing complication rates between the two groups.

This article was previously presented as a paper at the Fourth ISA YUVACON 2025 and 10th ISACON Annual Telangana State Conference, held in Hyderabad, Telangana, India, from April 25, 2025, to April 27, 2025.

## Materials and methods

Study design, ethical consideration, and trial registration

This randomized, double-blind, interventional trial was conducted in the Department of Anesthesiology and Critical Care at the Kalinga Institute of Medical Sciences, Pradyumna Bal Memorial Hospital, Kalinga Institute of Industrial Technology, Deemed to be University, Bhubaneswar, India, after obtaining written informed consent from all participants. The study period extended from June 2023 to February 2025. Ethical approval was obtained from the Institutional Ethics Committee (approval no.: KIIT/KIMS/IEC/1225/2023), and the trial was registered prospectively with the Clinical Trials Registry of India (#CTRI/2023/05/052352, dated May 08, 2023).

Eligibility criteria

Parturients aged between 18 and 40 years with American Society of Anesthesiologists physical status (ASA-PS) II and III, scheduled for elective lower-segment cesarean section (LSCS), were included in the study. Patients having any contraindication to SA, a history of chronic headache, a known spinal deformity, a prolapsed intervertebral disc, previous spinal surgery, a history of pelvic inflammatory disease, more than three lumbar puncture attempts, a history of PDPH in previous surgeries, and a BMI greater than 35 kg/m² were excluded from the study.

Sample size calculation

The sample size was calculated based on incidence data from Gurulingaswamy et al., which indicated PDPH incidences of 18% in the median group and 4% in the paramedian group [14]. At a 5% level of significance, 95% confidence interval, and 80% power, the minimum required sample size was calculated to be 166 participants (83 per group), assuming a 10% attrition rate.

Randomization, allocation concealment, and blinding

A computer-generated random sequence (block size 4) allocated participants to the median (group M) or paramedian (group P) approach. Allocation assignments were sealed in sequentially numbered opaque envelopes and opened immediately prior to the procedure by the anesthesiologist performing the block. To maintain blinding, the anesthesiologist who performed the spinal block did not participate in post-operative follow-up assessments. Patients were positioned and draped to prevent them from seeing the needle entry site; the approach used was concealed from them. The investigator performing all post-operative outcome assessments (PDPH and PSBA) remained blinded to group allocation throughout the study. Participants were asked not to inspect or discuss the needle site post-operatively, and routine wound care/dressing covered the region for the immediate post-operative period. Any instance of suspected unblinding (patient statement that revealed perceived approach or other disclosure) was recorded; no formal unblinding events occurred during follow-up.

Anesthetic management

All participants underwent a pre-operative evaluation one day before surgery, including routine laboratory tests and electrocardiography. Standard monitoring included continuous electrocardiography, non-invasive blood pressure measurement, and pulse oximetry. All participants underwent pre-operative evaluation and consent. On the day of surgery, patients were shifted to the pre-operative holding area, where an 18-gauge intravenous cannula was inserted in the non-dominant hand, and preloading with 15 mL/kg of Ringer’s lactate solution was initiated. SA was administered at L3-L4 using either the median or paramedian approach with 2.2 mL of 0.5% hyperbaric bupivacaine. All spinal anesthetics were performed with the patient in the sitting position on the operating table with feet supported and the back flexed. All spinal anesthetics were performed using a 25-gauge Quincke needle (BD Spinal Needle; Franklin Lakes, NJ: Becton Dickinson) together with a 20-gauge introducer, which was used consistently for both the median and paramedian approaches to minimize needle deflection and standardize technique. No ultrasound or other imaging modalities were used for spinal landmark identification; all procedures relied on anatomical surface landmarks. All procedures were performed by one anesthesiologist with more than 10 years of experience in obstetric regional anesthesia to minimize inter-operator variability. For each patient, the number of needle attempts was recorded, with an “attempt” defined as each new skin puncture. All procedures were completed within one attempt unless explicitly noted otherwise.

Paramedian Approach Technique

For participants randomized to the paramedian group, spinal anesthesia was performed using a standardized paramedian technique. The skin entry point was located approximately 1 cm lateral to the midline at the L3-L4 interspace. After 2 mL of 2% lignocaine was injected, a 25G Quincke spinal needle was inserted with a 10°-15° medial angulation toward the midline and a slight cephalad tilt of about 10°, moving toward the lamina. Once the lamina was contacted, the needle was walked cephalad into the interlaminar space until the ligamentum flavum was entered. The needle was then advanced slowly until free flow of cerebrospinal fluid was obtained, confirming intrathecal placement.

Median Approach Description

For the median group, the needle was inserted in the midline at the L3-L4 interspace with a perpendicular trajectory, applying minimal cephalad angulation as needed to traverse the interspinous ligament, ligamentum flavum, and dura.

Visual analog scale training and standardization

All patients were trained pre-operatively on the use of the visual analog scale (VAS) using a standardized 10-cm printed scale. The investigator explained the endpoints (“0=no pain” and “10=worst imaginable pain”) and demonstrated how to indicate pain intensity [15].

A physical VAS card was provided to each participant at discharge for consistency in interpreting pain severity at later time points. During follow-up telephone calls at one week, one month, and three months, patients were explicitly asked to refer to the same VAS card to ensure standardized scoring. If a patient no longer had the card, a verbal description of the 10-cm scale was repeated for standardization, and this information was noted in the follow-up record.

Outcome measures

Participants were monitored for the development of PDPH and PSBA post-operatively. PDPH was assessed at 24 h, 72 h, and one week, whereas PSBA was evaluated at one week, one month, and three months. Pain severity was assessed using the VAS. PDPH was diagnosed according to the International Classification of Headache Disorders criteria, characterized by an orthostatic headache that develops within seven days of dural puncture and resolves within 14 days or after an epidural blood patch [7]. PSBA was defined as localized lower back pain occurring after SA without other identifiable pathology [11]. The VAS was used to evaluate PSBA one week, one month, and three months after the procedure. VAS was used as the primary outcome measure because of its established validity for pain quantification; however, we acknowledge that PSBA may be influenced by factors beyond needle insertion, including surgical positioning, muscle strain, and duration of the cesarean procedure. These potential confounders were not quantified separately and may have contributed to patient-reported backache severity. The primary outcome was the incidence of PDPH at 24 h. The secondary outcomes included the incidence of PDPH at 72 h and one week, as well as PSBA at one week, one month, and three months.

Statistical analysis

All data entry and statistical analyses were performed using Microsoft Excel version 2019 (Redmond, WA: Microsoft Corp.). Data were analyzed using Statistical Package for the Social Sciences (SPSS) software, version 26.0 (Armonk, NY: IBM Corp.). Descriptive statistics were used to summarize demographic variables, with continuous data expressed as mean±standard deviation (SD) and categorical variables presented as frequencies and percentages. The normality of continuous data was assessed using the Shapiro-Wilk test. The independent samples t-test was used to compare groups for continuous variables that were normally distributed. The Mann-Whitney U test was used to compare groups for continuous variables that were not normally distributed. Categorical variables - including the incidence and severity of PDPH and PSBA - were analyzed using the chi-square test when all expected cell counts were ≥5 and Fisher’s exact test when any expected frequency was <5. The level of statistical significance was set at p<0.05 for all analyses.

## Results

**Figure 1:**
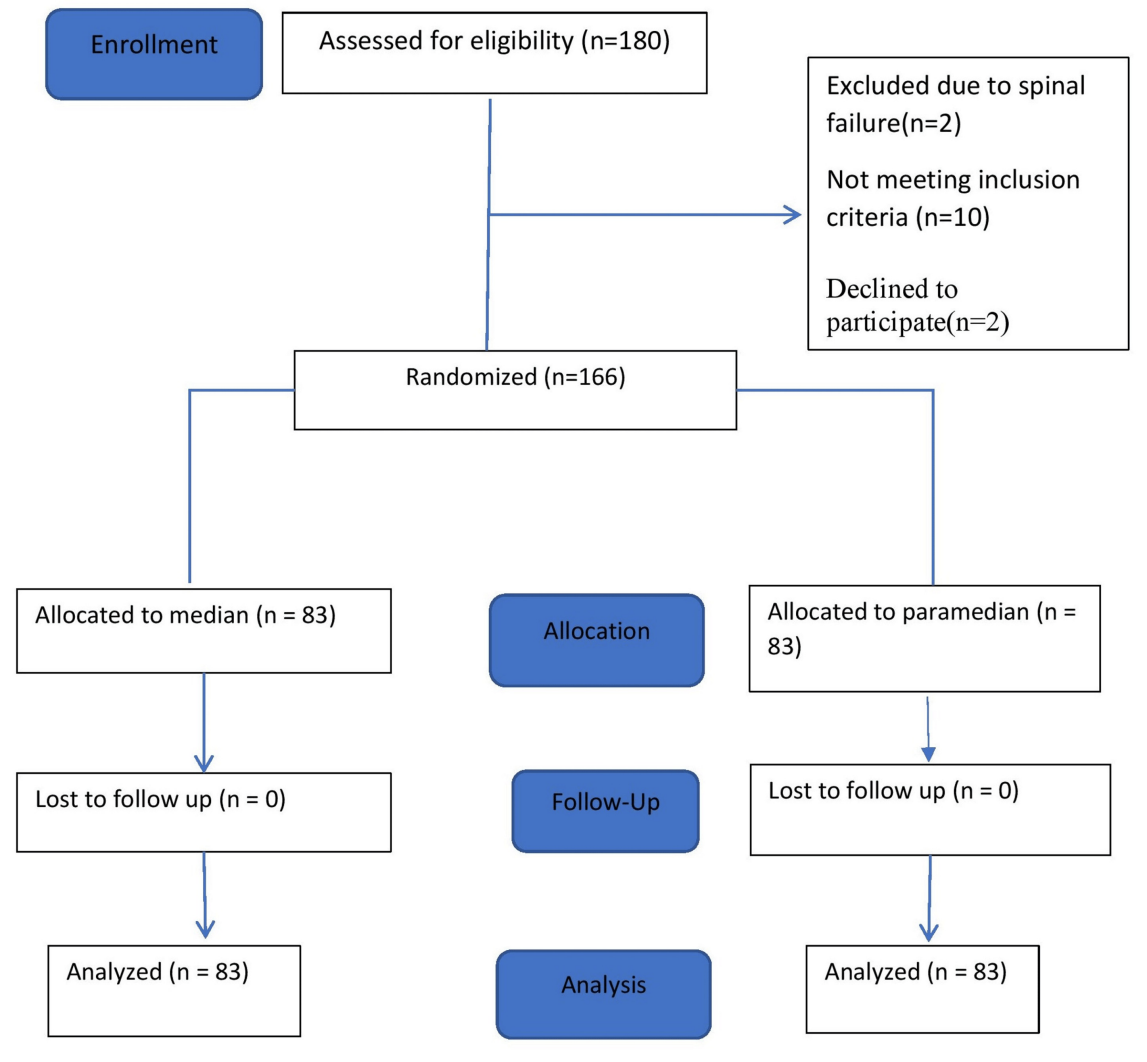
CONSORT flow diagram for enrolment, group allocation, follow-up, and analysis. CONSORT: Consolidated Standards of Reporting Trials

A total of 180 patients scheduled for elective cesarean section under SA were assessed for eligibility. Ten subjects were excluded for not meeting the inclusion criteria, which included morbid obesity (BMI ≥40 kg/m²), spinal deformity, and chronic headache disorders. Two were excluded due to spinal failure, and two declined to participate. Thereby, 166 subjects were randomized equally into the following two groups: group M (median approach, n=83) and group P (paramedian approach, n=83). There were no losses to follow-up in either group (Figure 1).

Baseline demographic characteristics, including age, weight, height, body mass index (BMI), ASA physical status, and gestational age distribution, were also similar across groups (Table 1). At 24 h post-operatively, PDPH was reported in 38 patients in group M and 13 patients in group P; the difference was statistically significant (p=0.001). At the 72-h follow-up, PDPH persisted in 22 patients from group M and 10 patients from group P (p=0.029). At one week, PDPH was present in 15 patients in group M and five patients in Group P (p=0.030) (Table 2).

**Table 1:** Baseline demographic and clinical characteristics of participants. Data are presented as mean±standard deviation (SD) or number (percentage). Continuous variables were compared using the independent t-test, and categorical variables were compared using the chi-square test. ASA: American Society of Anesthesiologists; BMI: body mass index

Variables	Paramedian (n=83)	Median (n=83)	p-Value
Age (years)	29.43±4.63	29.04±4.48	0.575
BMI (kg/m²)	26.36±3.37	26.67±3.43	0.560
Gestational age (weeks)	37.12±1.80	37.35±4.05	0.639
ASA physical status II, n (%)	67 (80.7%)	61 (73.5%)	0.283
ASA physical status III, n (%)	16 (19.3%)	22 (26.5%)	0.283

**Table 2:** Comparison of post-dural puncture headache (PDPH) in median and paramedian approach at different time intervals. *P<0.05 is considered significant. Data presented as numbers and percentages; analyzed using chi-square test or Fisher’s exact test as appropriate. PDPH: post-dural puncture headache

Time interval	PDPH status	Median (n=83)	Paramedian (n=83)	p-Value
24 h	Present	38 (45.8%)	13 (15.7%)	0.001*
	Absent	45 (54.2%)	70 (84.3%)	
72 h	Present	22 (26.5%)	10 (12.0%)	0.029*
	Absent	61 (73.5%)	73 (88.0%)	
One week	Present	15 (18.1%)	5 (6.0%)	0.030*
	Absent	68 (81.9%)	78 (94.0%)	

At 24 h, PSBA was observed in 29 patients in group M compared with 16 patients in group P. The difference between the two groups approached statistical significance (p=0.035). At one month, PSBA was present in 22 patients from group M and 15 patients from group P (p=0.263). At three months, PSBA persisted in 25 patients from group M and 17 patients from group P (p=0.211) (Table 3).

**Table 3:** Comparison of PSBA in median and paramedian approach at different time intervals. *P<0.05 is considered significant. Data presented as numbers and percentages; analyzed using chi-square test or Fisher’s exact test. PSBA: post-spinal backache

Time interval	PSBA status	Median (n=83)	Paramedian (n=83)	p-Value
One week	Present	29 (34.9%)	16 (19.3%)	0.035*
	Absent	54 (65.1%)	67 (80.7%)	
One month	Present	22 (26.5%)	15 (18.1%)	0.263
	Absent	61 (73.5%)	68 (81.9%)	
Three months	Present	25 (30.1%)	17 (20.5%)	0.211
	Absent	58 (69.9%)	66 (79.5%)	

**Figure 2:**
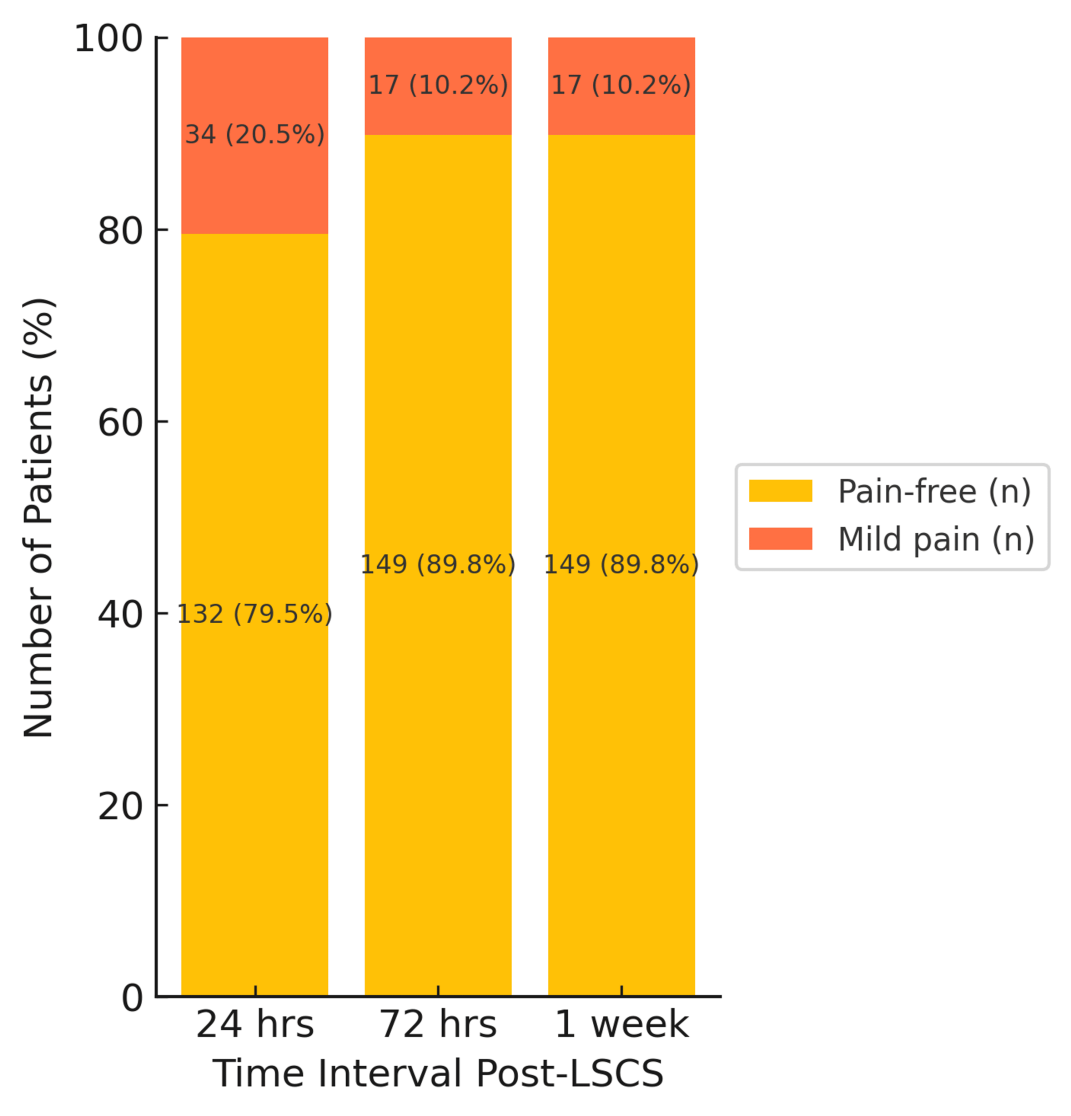
Distribution of severity of pain in PDPH at 24 h, 72 h, and one week. Each bar represents the number and percentage of patients experiencing mild, moderate, or no pain. Y-axis denotes number of patients (%). PDPH: post-dural puncture headache; LSCS: lower-segment cesarean section

The PDPH severity assessed by the VAS score declined steadily over time, with 132 patients pain-free at 24 h, increasing to 149 by one week. Mild pain decreased from 34 cases to 17, and no moderate or severe pain was reported throughout the follow-up period (Figure 2).

**Figure 3:**
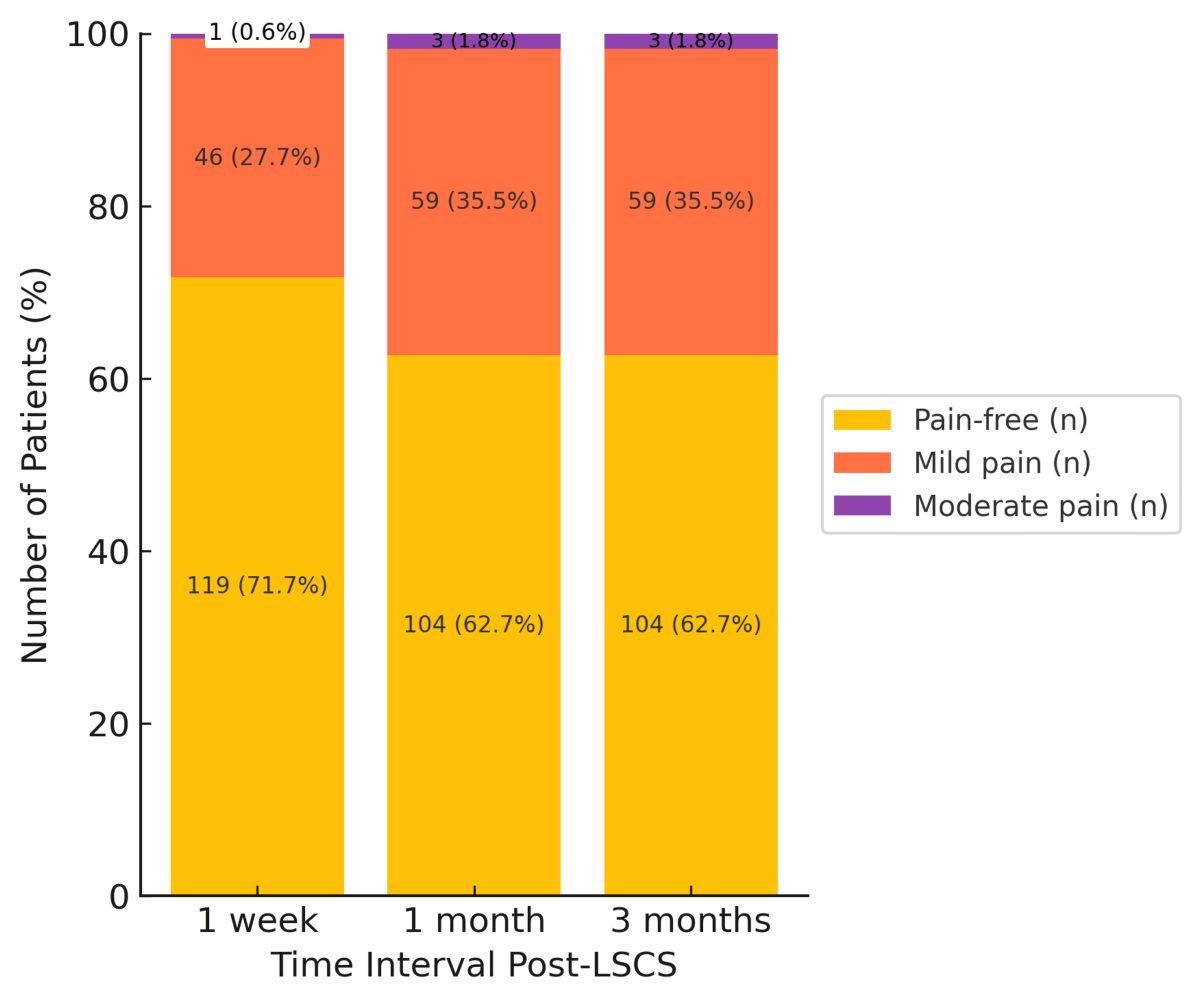
Distribution of severity of pain in PSBA at one week, one month, and three months. Each bar represents the number and percentage of patients with varying degrees of pain intensity. Y-axis denotes number of patients (%). PSBA: post-spinal backache; LSCS: lower-segment cesarean section

PSBA was generally mild, with most patients pain-free at all time points. At seven days, 119 patients reported no pain, 46 experienced mild pain, and one had moderate pain. By three months, the number of pain-free cases had decreased to 104, while the cases of mild pain increased to 59, and moderate pain was reported in three patients, with no severe cases observed (Figure 3).

Figures 2, 3 present combined severity distributions for the entire cohort. Although severity scores were compared between median and paramedian groups at each time point, no statistically significant between-group differences were observed; therefore, the graphical representations reflect overall patterns rather than group-specific curves.

## Discussion

This randomized double-blind trial compared the incidence and severity of post-dural puncture headache (PDPH) and post-spinal backache (PSBA) following the median and paramedian approaches to spinal anesthesia (SA) in parturients undergoing elective lower-segment cesarean section (LSCS). The findings demonstrate that the paramedian approach was consistently associated with lower incidence and severity of both PDPH and PSBA across multiple follow-up intervals. The differences in PDPH were statistically significant at 24 h, 72 h, and one week, whereas the reduction in PSBA reached significance at one week. These results illustrate the potential clinical advantage of the paramedian approach in minimizing post-operative discomfort after SA in obstetric patients.

The observed reduction in PDPH with the paramedian approach aligns with previous studies that have reported a protective effect from the needle’s more oblique trajectory through the dura [9,10,16]. This orientation probably causes a smaller dural rent and less cerebrospinal fluid (CSF) leakage than the direct perpendicular puncture of the median approach. Furthermore, the paramedian path bypasses midline ligamentous structures, potentially reducing tissue resistance and the number of needle redirections, which are known to increase PDPH risk [6,17]. Our findings, therefore, support the anatomical and physiological rationale that the paramedian route can decrease meningeal trauma and subsequent CSF loss, leading to a lower incidence of PDPH.

Anatomical and in vitro studies support this mechanism. Cadaveric and membrane-model experiments have shown that oblique dural punctures can create slit-like or flap-type defects rather than the circular holes produced by perpendicular midline entry, resulting in less cerebrospinal fluid egress. Scanning-electron microscopy studies similarly demonstrate that needle angle influences dural fiber separation and defect morphology, providing a plausible anatomical explanation for the lower PDPH rates observed with the paramedian approach [3,18].

The overall incidence of PDPH in this study is comparable to that reported in the literature for obstetric populations (1-14%), though our data show a distinct benefit favoring the paramedian approach [8]. Similar trends were reported by Wanjari et al. and Gurulingaswamy et al., who demonstrated significantly lower PDPH rates with the paramedian technique in patients undergoing cesarean section [14,16]. The magnitude of reduction observed in our study strengthens the argument for preferential use of the paramedian approach, particularly in parturients, where hormonal and anatomical factors may predispose them to higher PDPH rates.

The findings regarding PSBA are particularly noteworthy. Although post-operative backache following SA is generally benign and self-limiting, it can adversely affect early mobilization and maternal satisfaction [11]. In this study, PSBA incidence was significantly lower in the paramedian group at one week post-operatively, with a persistent downward trend at one and three months. However, the differences were not statistically significant. This indicates that tissue trauma, rather than dural irritation, is a significant factor in the onset of early PSBA. The lateral entry point of the paramedian approach bypasses the supraspinous and interspinous ligaments, structures often implicated in mechanical trauma and inflammation during the median approach [11,13]. Consequently, reduced ligamentous injury may account for the lower short-term back pain in our study population. Similar findings have been reported by Dadkhah et al. and Lee et al., reinforcing the notion that the paramedian technique may contribute to less post-operative musculoskeletal discomfort [12,13]. The early reduction in PSBA may suggest a role for reduced tissue trauma with the paramedian approach, but the result remains a hypothesis rather than a confirmed conclusion.

The rise in back pain noted at later follow-up intervals may suggest attribution bias, wherein patients associate naturally occurring postpartum musculoskeletal discomfort with their prior spinal anesthesia. Given that ligamentous relaxation, prolonged childcare posture, and core muscle deconditioning are common postpartum, these factors may confound PSBA reporting and inflate perceived technique-related pain.

Pain severity, as reflected by lower VAS scores in the paramedian group, further supports the hypothesis of reduced tissue and meningeal trauma. The consistent trend of milder pain across all follow-up intervals suggests that the paramedian technique not only reduces complication incidence but may also improve overall post-operative recovery and maternal comfort. Given that patient satisfaction is a key quality metric in obstetric anesthesia, this finding is of considerable clinical significance.

However, while our results favor the paramedian approach, not all previous studies have reported similar benefits. Bansal et al. and Kanagarajan et al. found comparable rates of PDPH between approaches, suggesting that factors such as operator skill, needle gauge, and patient anatomy may exert a greater influence than the choice of entry route alone [19,20]. The same senior anesthesiologist performed all procedures in our study to minimize variability, but we cannot entirely exclude subtle operator-dependent factors. These discrepancies across studies underscore the multifactorial nature of PDPH and PSBA and the need for multicenter trials to establish definitive recommendations.

Taken together, our findings support the clinical utility of the paramedian approach in reducing both PDPH and PSBA, particularly in populations predisposed to these complications, such as obstetric patients. From a practical perspective, the technique may be advantageous in cases of complex spinal anatomy, obesity, or limited interspinous space, where the median route can be challenging. By minimizing dural trauma and local tissue injury, the paramedian technique may enhance maternal recovery and satisfaction without compromising block efficacy.

Limitations

This study has several limitations. The study was conducted at a single tertiary care center, which may limit generalizability to other settings or patient populations. Although all procedures were standardized and performed by an experienced anesthesiologist, subtle variations in needle angulation and tissue resistance may have influenced the outcomes. Although every effort was made to maintain double-blinding (separate operator and blinded outcome assessor, draping, and allocation concealment), we acknowledge that perfect blinding in a procedural trial is difficult. Patients might at times perceive or infer the needle trajectory (e.g., differing sensations, comments from staff) or could inspect their back after the procedure, introducing potential attribution or observer bias. Pain assessment relied on patient-reported VAS scores, which are inherently subjective despite their widespread validity. Additionally, the follow-up period was restricted to three months; thus, long-term sequelae of PSBA could not be evaluated. This study did not assess postpartum mobility or maternal satisfaction, both of which may correlate with the severity or functional impact of post-spinal backache. Another limitation is that no objective musculoskeletal assessment or imaging (such as structured physical examination scoring, ultrasound, or MRI) was performed to differentiate procedure-related PSBA from unrelated postpartum musculoskeletal causes. This limits our ability to determine the precise etiology of backache reported during follow-up. Future multicenter studies with larger samples and extended follow-up are warranted to confirm these findings and explore underlying pathophysiological mechanisms.

## Conclusions

In this single-center randomized trial of parturients meeting our eligibility criteria, the paramedian approach was associated with a lower early incidence of post-dural puncture headache and reduced short-term post-spinal backache compared with the median approach. However, given the study’s single-center design, modest sample size, and the exclusion of specific higher-risk subgroups (for example, patients with prior PDPH or morbid obesity), these findings should be considered exploratory. They provide preliminary evidence warranting further investigation but, by themselves, do not support broad, practice-changing recommendations. Larger, multicenter studies including broader obstetric cohorts, with a focus on comparing various needle types and gauges within the paramedian technique, assessing long-term functional outcomes, and integrating ultrasound-guided methods, are needed to mitigate complications further.
